# Argan Callus Extract Restores Skin Cells via AMPK-Dependent Regulation of Energy Metabolism, Autophagy, and Inflammatory Pathways

**DOI:** 10.3390/antiox14070804

**Published:** 2025-06-28

**Authors:** Ramona Hartinger, Felix Quirin Fenzl, Vanessa Martina Nalewaja, Karima Djabali

**Affiliations:** Epigenetics of Aging, Department of Dermatology and Allergy, TUM School of Medicine, Munich Institute of Biomedical Engineering (MIBE), Technical University of Munich (TUM), 85748 Garching, Germany; ramona.hartinger@tum.de (R.H.);

**Keywords:** senescence, argan callus extract, skin-derived precursor cell, mesenchymal stem cells, autophagy, antioxidant, anti-aging

## Abstract

Skin aging is driven by cellular senescence, oxidative stress, and diminished regenerative capacity. In this study, we investigated the effects of PhytoCellTec™ Argan, an argan callus extract (PC), on primary human fibroblasts and adult stem cells. PC treatment (0.1% and 0.5%) significantly enhanced fibroblast proliferation, reduced senescence-associated β-galactosidase activity, and decreased the expression of p16, p21, and phosphorylated NFκB. PC treatment lowered intracellular ROS levels, increased ATP production, and promoted autophagy via LC3B-II accumulation and p62 reduction. In skin-derived precursor cells (SKPs), as well as mesenchymal stem cells (MSCs), PC treatment improved spheroid formation and growth while preserving the expression of key stemness markers, including Sox2, Oct4, and Nestin. Furthermore, PC exhibited antioxidant capacity (TEAC assay) and inhibited elastase, supporting its anti-aging potential. These findings suggest that PC is safe at concentrations below 1% and may serve as an effective natural compound to restore cellular homeostasis, reduce senescence and inflammation, and support stem cell health during aging.

## 1. Introduction

With an aging global population and a rising prevalence of age-related diseases, the focus on anti-aging mechanisms has intensified [1]. Consequently, research in this field is expanding rapidly, driven by its vast potential to enhance quality of life, advance cosmetic science, and deliver significant esthetic benefits [1]. As aging processes progress, the performance of tissues and organs declines due to the deterioration of metabolic, cellular, and physiological functions, leading to increased susceptibility in disease and mortality [2,3]. Age-related cellular alterations, known as the hallmarks of aging, include genomic instability, telomere attrition, epigenetic alterations, loss of proteostasis, deregulated nutrient sensing, mitochondrial dysfunction, cellular senescence, stem cell exhaustion, and altered intercellular communication [4]. These hallmarks represent cumulative damage and regulatory shifts that impair DNA repair, protein quality control, and metabolism [1,3,4]. As a result, cells lose their ability to maintain homeostasis, contributing to tissue degeneration and increased vulnerability to stress [3].

The skin is particularly susceptible to these cellular changes [5]. As the body’s largest organ and primary barrier to external stressors, the skin undergoes significant structural and functional alterations over time, influencing both its appearance and protective capabilities [5,6]. Serving as a protective barrier against pathogens, infections, chemicals, and mechanical impacts, the skin also plays a crucial role in preventing dehydration, regulating temperature, and enabling sensory perception [6,7,8]. Structurally, it consists of three distinct layers: the epidermis, dermis, and hypodermis [7]. The epidermis, the outer layer of the skin, is a dynamically renewed epithelium composed of multilayered keratinocytes, along with melanocytes, Langerhans cells, and Merkel cells [9,10,11]. Underneath it lies the dermis, separated by the basement membrane, which serves as the mesenchymal layer of the skin [10,12]. The dermis contains connective tissue rich in collagen and elastic fibers, along with lymphatic vessels, various fibroblast lineages, macrophages, nerve fibers, sweat glands, and hair follicles, providing mechanical strength, nutritional support, and vascular function [9,10,11]. The innermost layer, the hypodermis, consists of loose connective tissue, fat cells, and various cell types like fibroblasts, adipocytes, and macrophages [11]. This layer cushions against mechanical pressure and temperature extremes and plays a key role in energy storage and metabolic processes [9].

Although the skin undergoes continuous renewal [9], aging significantly impacts its functions, leading to wrinkles, dryness, thinning, altered pigmentation, and compromised barrier integrity [13,14]. Cellular changes are key contributors to the aging process as dermal cells lose their ability to proliferate and enter a state of replicative senescence [13,15]. Moreover, the skin’s self-renewing capacity is significantly diminished, further accelerating the visible effects of aging [13,15]. During the aging process, skin components like melanocytes, Langerhans cells, and the extracellular matrix undergo degeneration. Structural changes involve a reduction in dermal thickness, deterioration of the dermal–epidermal junctions, and alterations in connective tissue, including the breakdown of collagen and elastin fibers [13,14,15,16,17]. Fibroblast function is also significantly altered. During aging, these cells, which are crucial for maintaining tissue structure and repair, lose their ability to proliferate and produce essential extracellular matrix components. Furthermore, they develop a senescence-associated secretory phenotype (SASP) that fosters a pro-inflammatory environment [18,19]. These changes compromise tissue integrity and function, contributing to the overall decline in systemic health with age [18]. Epidermal stem cells, which maintain skin structure through self-renewal and tissue repair in healthy individuals, also experience age-related dysfunctions. Impaired stem cell mobilization or a reduced ability of stem cells to respond to proliferative signals further contribute to skin aging [20,21].

Consequently, it is a major goal to maintain fibroblast and stem cell function in the skin’s aging process in order to support skin homeostasis and repair mechanisms. Given the limitations of existing anti-aging and regenerative strategies, there is a pressing need for novel, effective, and natural compounds to support cellular rejuvenation and maintain cellular function during aging. Plant extracts, rich in bioactive molecules, have gained increasing attention for their potential to counteract age-related cellular dysfunction [22]. Among them, argan extract stands out due to its potent antioxidant and anti-inflammatory properties, positioning it as a promising candidate for maintaining cellular function and promoting healthy aging [23,24]. *Argania spinosa*, commonly known as argan, is an endangered species native to the arid regions of southwestern Morocco holding significant ecological and socioeconomic value [24].

Argan extracts contain a complex matrix of ingredients like salts, acids, phenols, sugars, proteins, and antioxidants, which contribute to the protection of skin stem cells [25,26,27,28]. Notably, its phenolic compounds, such as tocopherols (Vitamin E), serve as potent antioxidants to combat oxidative stress [26]. Additionally, *Argania spinosa* extracts also include triterpenoids with anti-inflammatory properties, along with essential fatty acids like oleic and linoleic acids, that help moisturize the skin, repair the skin barrier, and reduce inflammation [27,28,29,30,31]. Most studies investigating argan extract focus on its leaves, fruits or oil. However, as argan trees are rare, endangered, and designated as a UNESCO World Heritage of Humanity since 1999, sustainable alternative methods for argan use should be considered [24]. One such innovation is PhytoCellTec™ Argan (Mibelle Biochemistry), a preparation derived from *Argania spinosa* callus cells cultured in vitro. These plant stem cells are cultivated under controlled conditions to produce specific bioactive compounds [32].

This study explored the beneficial effects of PhytoCellTec™ Argan (PC) on aged fibroblasts and stem cells, aiming to restore cellular homeostasis, enhance stem cell identity and growth, and promote overall cellular vitality. PC treatment effectively boosted fibroblast proliferation, optimized cellular homeostasis, and notably reduced inflammation markers and cellular senescence in aged fibroblasts. While it did not enhance stem cell properties, the extract exhibited excellent biocompatibility, showing no cytotoxic effects and maintaining stemness. These results highlight the promising potential of PC as a safe and effective agent for supporting cellular health.

## 2. Materials and Methods

### 2.1. Cell Culture and Compound Treatment

The normal human primary dermal fibroblast cell lines, utilized in this study, were purchased from the Coriell Institute for Medical Research (Camden, NJ, USA) and the Progeria Research Foundation Cell and Tissue Bank: GM05757C (7-year-old male), GM05565A (3-year-old male), HGFDFN369 (33-year-old male), HGMDFN368 (31-year-old female), and GM01651C (13-year-old female).

For cultivation, primary dermal fibroblasts were kept in DMEM (Thermo Fisher—Gibco, Waltham, MA, USA, D6429) enriched with 15% fetal bovine serum (FBS; Thermo Fisher—Gibco, Waltham, MA, USA, 10270106), 1% L-glutamine (Thermo Fisher—Gibco, Waltham, MA, USA, 25030081), 0.5% gentamycin (Thermo Fisher—Gibco, Waltham, MA, USA, 15710049), and 1% penicillin/streptomycin (Thermo Fisher—Gibco, Waltham, MA, USA, 1514022) and maintained in a cell incubator (Binder, Tuttlingen, Germany; model 9140-0046) at 37 °C in 5% CO_2_ atmosphere. The cultures were subcultured at approximately 80% confluence, and the senescence level for all experiments ranged from 15 to 25%, with specific passage numbers detailed in Table 1. The young SKPs were isolated from early fibroblast cultures with a SNS level of approximatively 5%.

For the PC treatment, fibroblasts were grown in two different conditions: control medium without the extract (mock), or in a medium supplemented with the PC argan extract at a specific concentration. The PhytoCellTec™ Argan (PC) extract was obtained from Mibelle Biochemistry (Mibelle, Buchs, Switzerland, Lot #0530-010) in powder form and was directly dissolved in growth medium (DMEM + supplements). Mibelle Biochemistry (Buchs, Switzerland) has developed liposomal plant stem cell extracts for cosmetic applications, including PhytoCellTec™ Argan, which is derived from Argania spinosa callus cultures [33]. PhytoCellTec™ Argan (Mibelle Biochemistry, Buchs, Switzerland; Lot #0530-010) is a commercially available isomalt-based granulate composed of 0.8% *Argania spinosa* sprout cell culture extract (dry), 0.8% phospholipids, 93% isomalt, 0.3% sodium benzoate, and approximately 6% water [34]. For in vitro experiments, the extract was dissolved in sterile fibroblast growth medium (DMEM + supplements), aliquoted, and stored at −20 °C. In addition, the composition and manufacturing process are publicly disclosed in the international patent application WO2014167557A1, filed on January 30, 2014 by Erez Zabari and published on 16 October 2014 (PCT/IL2014/050102), which further supports the traceability and reproducibility of the product used [34].

All experiments were performed using the same production lot to ensure consistency and reproducibility.

### 2.2. Skin-Derived Precursor Cells and Stem Cells

For stem cell analyses, skin-derived precursor cells (SKPs) were isolated with low-pH isolation from primary dermal fibroblast cultures as detailed described by Budel et al. [35]. Once the primary fibroblast cultures reached approximately 80% confluence, they were collected using trypsin-EDTA (Thermo Fisher—Gibco, Waltham, MA, USA, 25200056). After centrifugation at 350× *g* for 5 min, the cell pellet was washed with PBS (Merck KGaA, Darmstadt, Germany, D8537) and 1 × 10^6^ cells were resuspended in 500 µL of Hank’s balanced salt solution (HBSS; Thermo Fisher—Gibco, Waltham, MA, USA, 14175053), which had been adjusted to a pH of 5.7 using HCl (Merck KGaA, Darmstadt, Germany, 1.00319.2500). For SKP isolation, this suspension was incubated at 37 °C for 25 min, with resuspending every 5 min. After incubation, the cells were pelleted at 350× *g* for 5 min and the pellet was then solved in 6 mL of SKP medium, containing 4:1 DMEM low glucose (Thermo Fisher—Gibco, Waltham, MA, USA, 21885025) to F12 (Thermo Fisher—Gibco, Waltham, MA, USA, 21765029), supplemented with 20 ng/mL EGF (Thermo Fisher—Gibco, Waltham, MA, USA, PHG0311), 40 ng/mL bFGF (Thermo Fisher—Gibco, PHG0026), 2% *v*/*v* B27 (Thermo Fisher—Gibco, Waltham, MA, USA, 17504044), 0.5 µg/mL fungizone (Thermo Fisher—Gibco, Waltham, MA, USA, 15290018), and 100 U/100 µg/mL penicillin/streptomycin (Thermo Fisher—Gibco, Waltham, MA, USA, 1514022). The suspension was equally divided into two T25 non-tissue-culture-treated flasks (Fisher Scientific—Falcon, Hampton, NH, USA, 10112732). To prevent the spheroids from adhering to the flask surfaces, the cultures were gently agitated daily by pipetting, and on day 2 and day 4 a 10× SKP supplement (SKP medium containing 10× EGF, bFGF, and B27) was added and diluted to a final concentration of 1× in the culture medium.

In addition, human mesenchymal stem cells (hMSCs) and bone marrow stem cells (BMSCs) were utilized in this study. The hMSCs were obtained from Sigma Aldrich (umbilical cord hMSCs (C-12971) and bone marrow-derived hMSCs (C-14090), Merck KGaA, Darmstadt, Germany) and cultured under the same conditions as the SKPs.

### 2.3. Senescence-Associated β-Galactosidase Assay

To assess cellular senescence (SNS) in fibroblast monocultures, the β-Galactosidase assay following the protocol described by Dimri et al. (1995) [36] was applied. Briefly, the fibroblasts were washed with PBS and then fixed for 5 min in a fixation solution containing PBS supplemented with 2% formaldehyde (Sigma-Aldrich, St. Louis, MO, USA, 104003) and 0.2% glutaraldehyde (Sigma-Aldrich, St. Louis, MO, USA, G5882). After fixation, the cells were washed twice with PBS and subsequently incubated overnight at 37°C (without CO_2_) in an SA-β-Gal staining solution. The staining solution included 2 mM MgCl_2_ (Sigma-Aldrich, Merck KGaA, Darmstadt, Deutschland, M-1028), 5 mM potassium ferrocyanide (Sigma-Aldrich, Merck KGaA, Darmstadt, Deutschland, P9387), 5 mM potassium ferricyanide (Merck KGaA, 104973, Darmstadt, Germany), 150 mM NaCl (Sigma-Aldrich, St. Louis, MO, USA, 310166), 0.5 mg/mL 5-bromo-4-chloro-3-indolyl β-D-galactoside (X-gal; Sigma-Aldrich, St. Louis, MO, USA, 3117073001), and 40 mM citrate/sodium phosphate buffer (pH 6.0) (Merck KGaA, Darmstadt, Deutschland). On average, approx. 1000 cells per sample were analyzed, with cells showing blue staining classified as senescent.

### 2.4. Determination of Cell Number, Cumulative Population Doubling, and Cytotoxicity

To assess the cell count and viability, fibroblasts were plated at a density of 3.5 × 10^3^ cells per well in 24-well plates (Sarstedt, Nümbrecht, Germany) and treated with various concentrations of PC or left without treatment as controls. After the treatment period, cells were detached by trypsin and analyzed with the Muse™ Cell Analyzer (Merck Millipore, Burlington, MA, USA) using the Muse™ Count and Viability Kit (Cytek^®^ Biosciences, Amsterdam, The Netherlands, SKU MCH100102). This assay is based on a dual-fluorescent dye system: acridine orange (AO), which stains all nucleated cells, and propidium iodide (PI), which selectively stains non-viable cells with compromised membranes. Live cells are identified as AO+/PI−, while dead cells are identified as AO+/PI+. This methodology facilitates precise discrimination between viable and non-viable cells.

Cytotoxicity was assessed by calculating the percentage of dead cells using the formula:

% dead cells = 100% − % viable cells

Cumulative population doubling (CPD) was identified by the following equation:

n = 3.32 (log cells harvested − log cells seeded) + x,

where n represents the CPD at the given culture stage, and x corresponds to the previous CPD value [37].

### 2.5. BrDU Staining

In addition to a cell number analysis, BrdU staining was performed on proliferating fibroblasts to assess the number of actively dividing cells. The thymidine analog 5-Bromo-2′-deoxyuridine (BrdU) is incorporated into newly synthesized DNA during the S-phase of the cell cycle, substituting for thymidine [38].

Fibroblasts, both treated and untreated, were cultured on glass coverslips and incubated for 40 min in culture growing medium containing 10 µM BrdU (B5002, Merck KGaA, Darmstadt, Germany). Cells were then fixed with 2% PFA (104005, Merck KGaA, Darmstadt, Germany) for 15 min and washed two times with PBS. To denature DNA, the coverslips were incubated in 2 M HCl (Merck KGaA, Darmstadt, Germany) for 1 h at RT, followed by three washes with PBS. Permeabilization was implemented using 0.2% Triton X-100 (Triton X-100, Merck KGaA, Darmstadt, Germany) in PBS for 10 min. After an additional washing step for 5 min with PBS, cells were blocked at RT with 10% FBS (Thermo Fisher—Gibco, Waltham, MA, USA, 10270106) in PBS for 40 min. Subsequently, they were incubated overnight at RT with an anti-BrdU antibody conjugated to a fluorescent dye (BrdU Monoclonal Antibody (MoBU-1), Alexa Fluor™ 488 (Thermo Fisher Scientific, B35130, 1:100). The next day, coverslips were washed three times with blocking buffer and once with PBS. Cells were then counterstained using DAPI in a Vectashield mounting medium (Vector Laboratories, Burlingame, CA, USA, VEC-H-1200), and the samples were analyzed under a BZ-X810 fluorescence microscope (KEYENCE DEUTSCHLAND GmbH, Neu-Isenburg, Germany).

### 2.6. Western Blot

For Western blot analyses, fibroblasts were washed once with PBS and then collected on ice by scraping in lysis buffer containing 150 mM NaCl (Merck KGaA, S7653, Darmstadt, Germany), 1% Triton (Triton X-100, Merck KGaA, Darmstadt, Germany), 1% SDS (SDS, Merck KGaA, Darmstadt, Germany), 1 mM EDTA (E9884, Merck KGaA, Darmstadt, Germany), 50 mM Tris (T9285, Merck KGaA, Darmstadt, Germany), 45% 2× Lämmli sample buffer (BioRad, Hercules, CA, USA, 1610737), 3% β-Mercaptoethanol (BioRad, 1610710), 0.5% Protease Inhibitor (Thermo Fisher, 78430), 0.5% Phosphatase Inhibitor (Thermo Fisher, 78426), and 200 mM PMSF (8553S, Cell Signaling, Leiden, The Netherlands). The total protein concentration was determined using the Bradford assay, with BSA (BSA; BioRad Laboratories, 5000206, Hercules, CA, USA) as the standard. Protein lysates (20 μg) were separated using SDS-PAGE in either an 8% or 15% gel and transferred onto nitrocellulose membranes (GE10600003, Sigma-Aldrich, St. Louis, MO, USA) via the wet-transfer method. The membranes were blocked with 5% non-fat milk (Merck KGaA, Darmstadt, Germany) for 1 h at room temperature (RT) and subsequently incubated overnight at 4 °C with primary antibodies targeting various proteins, including p62 (SQSTM1, 66184-1-Ig, Proteintech, Manchester, UK, 1:1000), p16 (INK4A, JC8, sc-56330, Santa Cruz, Dallas, TX, USA, 1:1000), p21 (Wafa1/Cip1, DCS60, Cell Signaling, Leiden, The Netherlands, 1:1000), LC3B (LC3A/B, D3U4C, 12741, Cell Signaling, Leiden, The Netherlands, 1:2000), P-AMPK (Phospho-AMPKα, Thr172, 40H9, Cell Signaling, Leiden, The Netherlands, 1:1000), AMPK (AMPKα, Cell Signaling, Leiden, The Netherlands, 1:1000), P-NFκB (Phospho-NF-κB p65, Ser536, 93H1, Cell Signaling, Leiden, The Netherlands, 1:2000), NFκB (NF-κB p65, D14E12, Cell Signaling, Leiden, The Netherlands, 1:2000), and GAPDH (G9545, Sigma-Aldrich, St. Louis, MO, USA, 1:5000). Following incubation, the membranes were washed three times with TBS-Tween (Tween Merck KGaA, Darmstadt, Germany), and afterwards incubated for 1 h at RT with horseradish peroxidase-conjugated secondary antibodies (Jackson ImmunoResearch Laboratories, West Grove, PA, USA) specific to either rabbit (1:5000) or mouse (1:5000). After additional TBS-Tween washes, signal detection was performed using luminol-enhanced chemiluminescence (BioRad, Hercules, CA, USA, 1705061), and the bands were visualized with the ChemiDoc™ MP imaging system (BioRad, Hercules, CA, USA). Image quantification was conducted with ImageJ software (version 1.53f51, Java 1.8.0_172, developed by Wayne Rasband and contributors from the National Institutes of Health, Bethesda, MD, USA), with normalization to GAPDH as an internal control.

### 2.7. Cryopreservation and Cryosectioning

For further analyses, the stem cell spheres were cryopreserved by embedding in optimal cutting medium (OCT) and cryosectioning. Cryosection molds (Merck KGaA, Darmstadt, Germany) were prepared on ice with OCT (Tissue-Tek^®^ O.C.T.™ Compound, SienceServices, Munich, Germany, SA62550) and freezing solution was set up by mixing 30% sucrose solution (Merck KGaA, Darmstadt, Germany) in PBS with OCT at a ratio of 1:2 to ensure proper preservation during freezing. Next, the SKPs were harvested into a 15 mL tube (Sarstedt, Nümbrecht, Germany), centrifuged at 350× *g* for 5 min and washed twice with 5 mL PBS. Afterwards, the cell pellet was resuspended in 30 µL of the freezing solution and small droplets (10–15 µL) of the SKP suspension were then placed into the center of each OCT-filled cryomold. The samples were immediately snap-frozen in liquid nitrogen and subsequently stored at −80 °C.

Cryosections were prepared using the Leica CM3050S cryostat (Leica, Wetzlar, Germany), with both the sectioning head and the chamber maintained at a constant temperature of −25 °C. SKPs embedded in OCT were cut into 5 µm thick sections and immediately mounted onto superfrost microscope slides (Fisher Scientific, Waltham, MA, USA, 15438060). The slides were allowed to air-dry, and stored at −80 °C.

### 2.8. Immunocytochemistry

For immunocytochemistry, the sections were fixed in 2% PFA (Merck KGaA, Darmstadt, Germany, 104005) for 10 min. After fixation, the slides were washed with PBS and cells were permeabilized using 0.2% Triton X-100 (Merck KGaA, Darmstadt, Germany, 104005) in PBS for 5 min, followed by an additional washing step with PBS for 5 min. Subsequently, fibroblasts were blocked at RT for 30 min in PBS containing 10% FBS (FBS; Thermo Fisher—Gibco, 10270106). The sections were then incubated overnight at 4 °C with the following primary antibodies: mouse anti-Sox2 (sc-365823, Santa Cruz, Dallas, TX, USA, 1:50), rabbit anti-Vimentin (ab137321, Abcam, Cambridge, UK, 1:1200), mouse anti-Nestin (sc-23927, Santa Cruz, Dallas, TX, USA, 1:50), rabbit anti-Fibronectin (15613-1-AP, Proteintech, Manchester, UK, 1:2000). Afterwards, the sections were washed four times with blocking buffer before being incubated for 1 h at RT with secondary antibodies conjugated to Alexa Fluor^®^ 488 or 555 (Life Technologies, Thermo Fisher, Waltham, MA, USA; A21202 anti-mouse-488 and A31572 anti-rabbit-555, 1:1000). After two additional washes with blocking buffer and two with PBS, the cells were counterstained using DAPI-containing Vectashield mounting medium (Vector Laboratories, Burlingame, CA, USA, VEC-H-1200). Fluorescence microscopy images were acquired using a Keyence BZ-X810 fluorescence microscope (KEYENCE DEUTSCHLAND GmbH, Neu-Isenburg, Germany).

### 2.9. Measurement of ROS

Intracellular reactive oxygen species (ROS) levels in live fibroblasts, with and without treatment, were quantified using the DCFDA—Cellular ROS Assay Kit/Reactive Oxygen Species Assay Kit (ab113851, Abcam, Cambridge, UK). The process is based on the use of 2′,7′-dichlorofluorescin diacetate (DCFDA), a cell-permeable fluorogenic dye. Once inside the cell, DCFDA is deacetylated by intracellular esterases to a non-fluorescent intermediate, which is then oxidized by ROS to form 2′,7′-dichlorofluorescein (DCF), a fluorescent compound. Fibroblasts were seeded in 96-well plates (Sarstedt, Nümbrecht, Germany) at a density of 2.5 × 10^4^ cells per well and allowed to adhere overnight. After washing with assay buffer, cells were incubated with 25 μM DCFDA for 30 min at 37 °C. Following another wash, fluorescence intensity was measured using a FLUOstar Omega Microplate Reader (BMG Labtech, Ortenberg, Germany) with excitation and emission wavelengths set at 485 nm and 520 nm, respectively. All experimental conditions were performed in triplicate for each cell line and treatment group.

### 2.10. Autophagy Activity

Autophagic activity in fibroblasts, with and without PC treatment, was evaluated using the Autophagy/Cytotoxicity Dual Staining Kit (Cayman Chemical Company, Ann Arbor, MI, USA). Autophagic vacuoles were stained with monodansylcadaverine (MDC), a fluorescent dye that selectively accumulates in multilamellar structures. Primary fibroblasts were seeded in 96-well plates (Sarstedt, Nümbrecht, Germany) at a density of 5 × 10^4^ cells per well and allowed to adhere overnight. Cells were then incubated with MDC (1:100 dilution) for 10 min at 37 °C, followed by a single wash with assay buffer. The fluorescence intensity of MDC-labeled autophagic vacuoles was measured using a FLUOstar Omega Microplate Reader (BMG Labtech, Germany) with excitation at 355 nm and emission at 520 nm. Each condition was analyzed in triplicate for every cell strain and treatment group.

### 2.11. Measurement of Cellular ATP

Adenosine triphosphate (ATP) levels were quantified using the CellTiter-Glo^®^ Luminescent Cell Viability Assay Kit (Promega, Madison, WI, USA). This assay measures ATP as an indicator of metabolically active cells, producing a luminescent signal proportional to ATP concentration. A total of 5 × 10^4^ treated and untreated cells were plated in triplicate in 96-well plates (Sarstedt, Nümbrecht, Germany) and incubated overnight to facilitate adherence. Afterwards, the fibroblasts were incubated with 100 µL of CellTiter-Glo reagent for 10 min, and luminescence intensity was recorded. An ATP standard curve was included for reference. All measurements were performed in a minimum of three separate independent experiments as triplicates.

### 2.12. Antioxidant ABTS Assay

The ABTS assay was conducted to assess the antioxidant capacity of the PC extract. First, ABTS was oxidized by adding 88 μL of a 200 mM potassium persulfate solution (Merck KGaA, Darmstadt, Germany, 216224) to 5 mL of a 7 mM ABTS solution (Merck KGaA, Darmstadt, Germany, A1888). This mixture was allowed to react overnight at RT, protected from light, to reach a stable oxidative state. The ABTS working solution was then prepared by diluting the oxidized ABTS solution in a 20 mM sodium acetate buffer (pH 4.5) until an absorbance of 0.700 ± 0.020 at 734 nm was achieved. To evaluate antioxidant activity, 188 μL of the ABTS working solution was added to each well of a microplate (Sarstedt, Nümbrecht, Germany), followed by 12 μL of the diluted sample or blank. The plate was incubated at RT for 30 min, protected from light, and absorbance was measured at 734 nm. For quantification, a standard curve was generated using TROLOX (Merck KGaA, Darmstadt, Germany, A1888) at various concentrations, with water as the blank. The ABTS assay measured the ability of the samples to scavenge ABTS radicals, and the inhibition percentage was determined using the following formula:

$$\%\;inhibition\;Sample=\frac{Abs\;(734\;nm)Blank-Abs\;(734\;nm)Sample}{Abs\;(734\;nm)Blank}\times 100$$

The antioxidant capacity was assessed as TROLOX Equivalent Antioxidant Capacity (TEAC) and reported in millimoles of TROLOX per gram of dry weight of the sample (mmol TROLOX/g dw).

### 2.13. Elastase Inhibition Assay

To determine the percentage of elastase inhibition, 50 µL of the PC extract was mixed with 160 µL of 0.20 mM Tris-HCl buffer (pH 8) and 20 µL of 0.80 mM N-Succinyl-Ala-Ala-Ala-p-nitroanilide (Merck KGaA, Darmstadt, Germany, S4760) substrate, prepared in Tris-HCl buffer. The solution was incubated for 10 min at RT. Subsequently, 20 µL of 1 U/L elastase enzyme (Merck KGaA, Darmstadt, Germany, E7885), prepared in Tris-HCl buffer, was added and the reaction was incubated for 20 min at RT. Absorbance was then measured at 410 nm using a spectrophotometer. Tris-HCl buffer was used as the negative control and showed no significant elastase inhibition.

The percentage of elastase inhibition was calculated using the following equation:

$$\%\;inhibition\;Sample=\frac{Abs\;(410\;nm)Negative\;Control-Abs\;(410\;nm)Sample}{(410\;nm)Negative\;Control}\times 100$$

### 2.14. Gene Expression Analysis

RNA was extracted using the GeneJET RNA Purification Kit (Thermo Fisher, Waltham, MA, USA) from fibroblast and stem cell pellets. The RNA concentration was determined with a NanoDrop ND-1000 spectrophotometer (Thermo Fisher, Waltham, MA, USA). For cDNA synthesis, 500 ng of RNA was reverse-transcribed using the High-Capacity cDNA Reverse Transcription Kit (Thermo Fisher, Waltham, MA, USA). Real-time PCR primers were designed via NCBI/Primer-BLAST and procured from Thermo Fisher (Waltham, MA, USA) [39]. A detailed list of the quantified genes and their corresponding primers is available in Table S1.

Quantitative real-time PCR was performed using the StepOnePlus™ Real-Time PCR System (Thermo Fisher, Waltham, MA, USA) with PowerUp™ SYBR™ Green Master Mix (Applied Biosystems™, Thermo Fisher, Waltham, MA, USA). Each reaction mixture (20 µL total volume) contained 300 nM of each primer and 2.5 ng of template DNA. The thermal cycling conditions included an initial denaturation at 95 °C for 20 s, followed by 45 cycles consisting of 3 s of denaturation at 95 °C and 30 s of annealing/extension at 60 °C. Fluorescence signals were recorded between cycles 10 and 40. GAPDH was used as an internal reference gene, and all experiments were conducted in triplicate with three biological replicates.

### 2.15. Statistical Evaluation and Graphics

Three biological replicates were cultured per cell strain and analyzed under the respective treatment conditions. A total of 1000 cells were counted for both senescence and BrdU assessments.

The results are presented as mean values ± standard deviation (mean ± SD). Statistical comparisons between two groups were performed using students *t*-test and two-way ANOVA. Data analysis and visualization were conducted with GraphPad Prism (Version 6.01, GraphPad, Boston, MA, USA). Statistical significance was expressed as follows: ns (not significant, *p* > 0.05); * *p* ≤ 0.05; ** *p* ≤ 0.01; *** *p* ≤ 0.001; **** *p* ≤ 0.0001.

## 3. Results

### 3.1. Optimization of Effectiveness of Working Concentrations

Fibroblast cultures with a senescence level between 15 and 25% were treated without and with the PhytoCell^TM^ (PC) extract at concentrations of 0.025%, 0.05%, 0.1%, 0.25%, 0.5%, 1%, and 5%. On day 8 of cultivation, cell counts were performed using the Muse™ Cell Analyzer (Cytec Biosciences, Amsterdam, The Netherlands) to determine the cumulative population doubling (CPD) and cell viability. Additionally, senescence (SNS) levels were evaluated using the senescence-associated β-galactosidase (SA-β-gal) assay [36]. To assess cell proliferation, BrdU staining was performed at day 8 of treatment, where fibroblasts were stained with 10 µM BrdU, and BrdU-positive cells were quantified.

**Figure 1:**
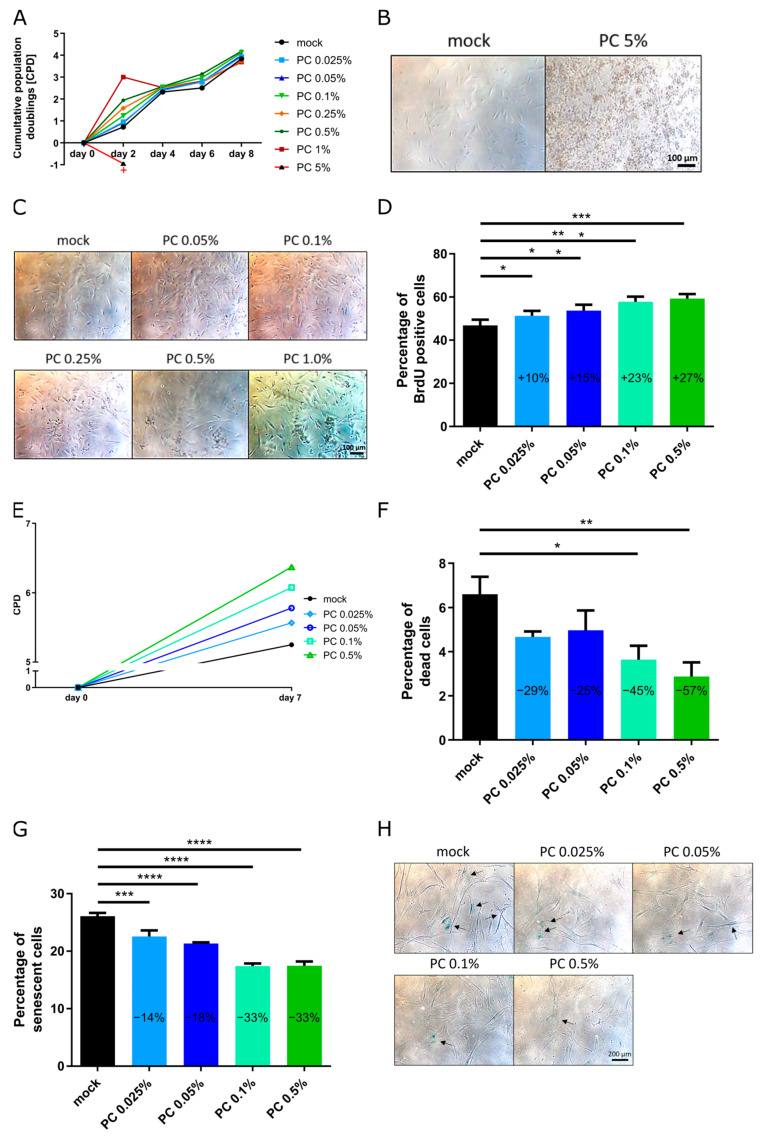
Determination of working concentration of PC. Fibroblasts were treated for 7–8 days with and without PC extract. (**A**) Cumulative population doubling to determine working concentrations. + marks cell death at the concentration PC 5% at day 2. (**B**) Representative brightfield images of fibroblasts treated 24 h without and with 5% PC. Scale bar: 100 µm. (**C**) Representative brightfield images of fibroblasts treated for 8 d without and with 0.05%, 0.1%, 0.25%, 0.5%, and 1% PC. Scale bar: 100 µm. (**D**) Percentage of BrdU-positive cells. (**E**) CPD at day 0 and day 7 with PC concentrations of 0.025%, 0.05%, 0.1%, and 0.5% and mock treatment. (**F**) Percentage of dead cells. (**G**) Percentage of senescent cells at day 8 of treatment. (**H**) SA-β-gal test performed using fibroblasts treated 8 d without and with PC concentrations of 0.025%, 0.05%, 0.1%, and 0.5%. Scale bar: 200 µm. Arrows show blue stained cells indicating cellular senescence. (**D**,**F**,**G**) Values are presented as mean ± SD (*n* = 3); * *p* < 0.05; ** *p* < 0.01; *** *p* < 0.001; **** *p* < 0.0001; assessed using one-way ANOVA.

Initially, fibroblasts were treated with PC at concentrations of 0.05%, 0.1%, 0.25%, 0.5%, 1%, 5% for 8 days (Figure 1). However, treatment with PC 5% resulted in complete cell death within 24 h, leading to its exclusion from further analyses (Figure 1A,B). The remaining concentrations (0.05%, 0.1%, 0.25%, 0.5%, and 1%) exhibited no cytotoxic effects, with the number of dead cells lower than that observed in the mock-treated control group (Figure 1A,C; Supplementary Figure S1). Cumulative population doubling (CPD) was increased across all tested concentrations (0.05%, 0.1%, 0.25%, 0.5%, and 1%), with the most pronounced effects observed at PC 0.1% and PC 0.5% (Figure 1A). Senescence (SNS) levels on day 8 of treatment were significantly reduced at all concentrations below 0.5%. However, PC 1% led to a significant increase in SNS levels by approximately 6% (Supplementary Figure S1). These findings indicate that PC concentrations below 0.5% effectively enhance fibroblast proliferation while mitigating senescence. PC was well tolerated at concentrations up to 0.5%, while higher concentrations (≥1%) induced cytotoxicity or increased senescence, underlining the concentration-dependent nature of the extract’s biological effects.

Based on these results, the working concentrations selected for further analysis were 0.025%, 0.05%, 0.1%, and 0.5%, and fibroblasts were treated for 7 days with and without the PC extract (Figure 1). Cell proliferation (BrdU staining), CPD, SNS, and cytotoxicity (percentage of dead cells) were assessed (Figure 1D–H). All concentrations significantly enhanced cell proliferation, as indicated by an increased percentage of BrdU-positive cells and higher CPD compared to the mock-treated group (Figure 1A,D–H). The most pronounced effects were observed with PC 0.1% and PC 0.5%. No cytotoxic effects were detected, and treatment with PC 0.1% and PC 0.5% even reduced the percentage of dead cells by half (Figure 1F). Additionally, SNS levels were significantly reduced across all treatments, with the greatest reductions observed at PC 0.1% (−8.9%) and PC 0.5% (−8.6%) (Figure 1G,H). Given their positive effects on enhancing proliferation and reducing senescence in aged fibroblasts, PC 0.1% and PC 0.5% were selected for further investigation.

### 3.2. Functional Characterization and Biological Effects

**Figure 2:**
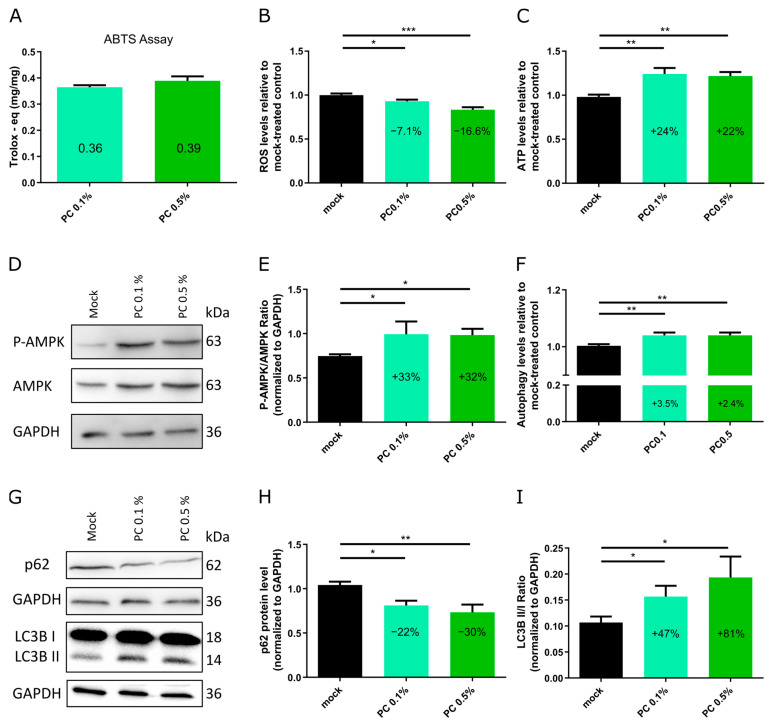
Effects of PC on aged fibroblasts. Fibroblasts were treated for 7 days with and without PC extract in 0.1% and 0.5% concentrations. (**A**) ABTS/Trolox equivalent antioxidant capacity (TEAC) of the PC extract in mg/mg. (**B**) Cellular ROS levels relative to mock-treated control. (**C**) Cellular ATP levels relative to mock-treated control. (**D**) Representative images of Western blot analyses for phosphorylated and total form of AMPK. (**E**) Ratio of phosphorylated to total AMPK protein level. (**F**) Autophagy levels analyzed by measuring monodansylcadaverine (MDC) via fluorescence photometry. (**G**) Representative images of Western blot analyses for p62 and LC3B protein levels. (**H**) p62 protein level normalized to GAPDH. (**I**) Ratio of LC3B-II to LC3B-I. (**A**–**C**,**E**,**F**,**H**,**I**) Graphs present mean ± SD (*n* = 3); * *p* < 0.05; ** *p* < 0.01; *** *p* < 0.001; assessed using one-way ANOVA.

To assess the effects of PC on cellular functions and related pathways, fibroblasts with a baseline senescence level of 15–25% were treated with PC 0.1% and PC 0.5% for 7 days. On day 7, cellular ROS, ATP, and autophagy levels were analyzed and Western blot analyses for p62, LC3B, and AMPK were performed (Figure 2). In addition, an ABTS (2,2-azinobis-(3-ethylbenzothiazoline-6-sulfonate) assay, also known as a Trolox equivalent antioxidant capacity (TEAC) assay, for determining the antioxidant capacity of the PC extract was conducted according to the manufacturer’s protocol (Merck KGaA) (Figure 2A).

The ABTS/TEAC of the PC extract was determined by measuring its ability to neutralize ABTS radicals. This colorimetric assay provides a quantitative evaluation of a compound’s free radical scavenging activity, indicating its potential to mitigate oxidative stress [40]. The PC extract showed antioxidant activity with a TEAC of 0.36 mg/mg (PC 0.1%) and 0.39 mg/mg (PC 0.5%), indicating its strong antioxidative effect (Figure 2A).

ROS levels are known to increase during the aging process, contributing to cellular damage and dysfunction [41,42]. Both treatments reduced cellular ROS levels compared to the mock-treated group, with PC 0.1% decreasing ROS by 7.1% and PC 0.5% by 16.6% (Figure 2B). These findings highlight the antioxidant properties of PC in fibroblast cultures, suggesting its potential to mitigate oxidative stress in aging cells.

ATP is crucial for cellular processes such as signaling, biosynthesis, and apoptosis regulation [43]. Both PC treatments significantly increased ATP levels, with enhancements of 24% and 22% for PC 0.1% and PC 0.5%, respectively (Figure 2C). Moreover, AMP-activated protein kinase (AMPK) is a key regulator of cellular energy homeostasis and plays a crucial role in inflammation-related pathways [44,45]. Therefore, its activity level was assessed by determining the protein levels of phosphorylated and total AMPK in aged fibroblasts (SNS 15–25%) by Western blotting. PC treatment activated AMPK, as evidenced by the increased P-AMPK/AMPK ratio compared to mock-treated counterparts (Figure 2D,E). Specifically, 0.1% PC elevated the P-AMPK/AMPK ratio by 33%, and 0.5% PC 0.5% by 32% (Figure 2E). These findings suggest that PC improves cellular energy balance, potentially by improving mitochondrial function as indicated by the increased ATP and reduced ROS in PC treated fibroblasts (Figure 2B,C).

Since the PC treatment induced the activation of AMPK signaling, which is known to inhibit mTOR activity, the master negative regulator of autophagy, it was hypothesized that PC treatment would consequently enhance autophagy [46]. Autophagy is a vital cellular process that maintains homeostasis by degrading and recycling cellular components, supporting energy production, and mitigating stress [47]. However, autophagy efficiency declines with age, leading to the accumulation of damaged components and organelles and increased cellular stress [47,48,49]. The PC treatment moderately enhanced autophagy, with increases of 3.5% at 0.1% PC and 2.4% at 0.5% PC (Figure 2F). To further assess autophagy flux, Western blot analyses were performed to determine the levels of autophagy markers p62 and LC3B (Figure 2G–I). p62 serves as an indicator of autophagic cargo degradation, while an increase in the LC3B II/I ratio indicates enhanced autophagosome formation [50,51]. The PC treatment resulted in a reduction in p62 protein levels by 22% (PC 0.1%) and 30% (PC 0.5%), accompanied by an increase in the LC3B II/I ratio by 47% (PC 0.1%) and 81% (PC 0.5%) (Figure 2H,I). These findings suggest that PC enhances autophagic flux, which may contribute to the attenuation of age-related cellular damage.

**Figure 3:**
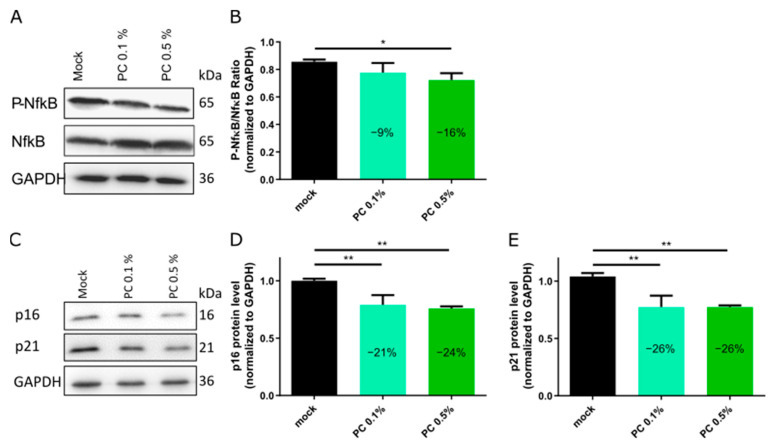
Western blot analyses for NFκB, p16, and p21 protein levels in fibroblasts treated for 7 days with and without PC extract. (**A**) Representative images of Western blot analyses for phosphorylated and total form of NFκB. (**B**) Ratio of phosphorylated to total NFκB protein level. (**C**) Representative images of Western blot analyses for p16 and p21. (**D**,**E**) Quantification of p16 and p21 protein levels normalized to GAPDH. (**B**,**D**,**E**) Graphs present mean ± SD (*n* = 3); * *p* < 0.05; ** *p* < 0.01; assessed using one-way ANOVA and Student’s unpaired *t*-test.

Given the interplay between impaired autophagy, mitochondrial dysfunction, and chronic inflammation in aging, we next investigated whether PC treatment could also modulate inflammatory signaling and cellular senescence pathways. For this, fibroblasts with a senescence level of 15–25% were treated for 7 days with or without PC at concentrations of 0.1% and 0.5%. On day 7, cell lysates were collected and Western blot analyses were performed to assess the expression of key markers of inflammation and senescence, including NF-κB, P-NF-κB, p16, and p21 (Figure 3). NF-κB, a key regulator of immune and stress responses, is activated with age, contributing to chronic low-grade inflammation and age-related cellular damage [44,45]. Treatment with PC resulted in a concentration-dependent reduction in NF-κB activity, as indicated by a decrease in P-NF-κB/NF-κB ratio by 9% at 0.1% PC and 16% at 0.5% PC (Figure 3A,B). These results suggest that PC reduces pro-inflammatory signaling.

Given the critical role of inflammation in promoting cellular senescence [18], we further examined the expression of senescence markers. p16 and p21 are key regulators of cellular senescence (SNS), contributing to irreversible cell cycle arrest and accumulation of senescent cells with age [4,18,51]. PC treatment reduced the expression of both p16 and p21 (Figure 3C–E), corroborating with the decreased number of senescence cells (Figure 1G). Specifically, p16 levels were reduced by 21% with 0.1% PC and by 24% with 0.5% PC, while p21 levels decreased by 26% at both concentrations (Figure 3C–E). These findings suggest that PC mitigate cellular senescence, potentially reducing pro-inflammatory factors characteristic of the senescence-associated secretory phenotype (SASP) [52].

Collectively, 0.1% and 0.5% PC treatments exert multi-cytoprotective effects that promote cellular health and resilience. Specifically, PC acts as an effective antioxidant by reducing intracellular ROS levels and enhancing mitochondrial function, as indicated by increased ATP production. Furthermore, PC improved autophagy efficiency, reduced NF-κB activity, and mitigated cellular senescence by decreasing the expression of key senescence markers, p16 and p21. These combined effects suggest that PC treatment improves cellular homeostasis and may delay age-related cellular dysfunction.

### 3.3. Effect of PhytoCell on Human Adult Stem Cells

Having established the beneficial effects of PC on dermal fibroblasts, we next investigated its impact on stem cells, which are essential for tissue regeneration and the long-term maintenance of skin integrity. The skin undergoes continuous renewal, making stem cell homeostasis essential for sustaining regenerative capacity throughout aging.

To assess the effects of PC treatment on stem cell biology, we applied PC to three distinct stem cell models: skin-derived precursor cells (SKPs), umbilical cord-derived mesenchymal stem cells (hMSCs), and human bone marrow-derived stem cells (BMSCs). This approach allowed us to explore PC’s potential in modulating stem cell function and broader implications for cellular health.

**Figure 4:**
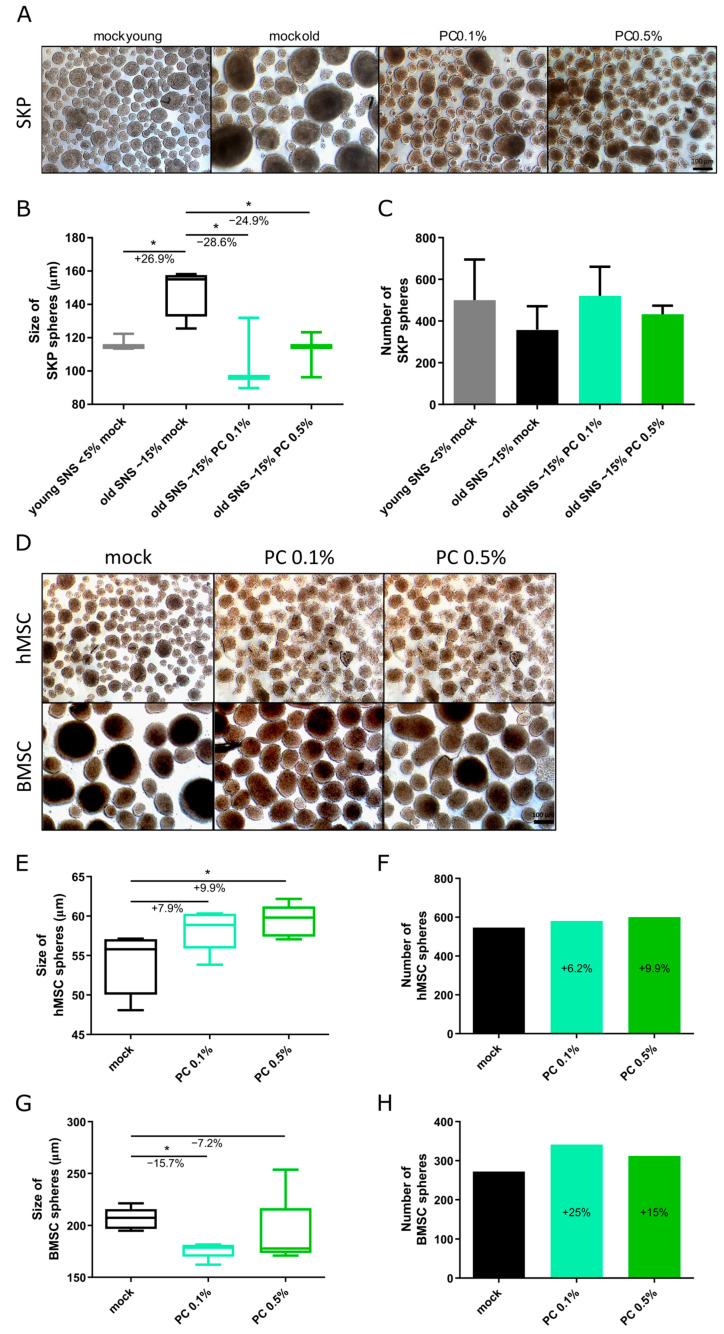
Effect of PC on adult human stem cells at day 5 of cultivation with and without PC treatment (0.1% and 0.5%). (**A**) Representative images of young and old SKPs. Scale bar: 100 µm. (**B**) Size of young and old SKPs. (**C**) Number of young and old SKPs. (**D**) Representative images of hMSCs and BMSCs. Scale bar: 100 µm. (**E**,**G**) Size of hMSCs (**E**) and BMSCs (**G**). (**H**,**F**) Number of hMSCs (**F**) and BMSCs (**H**). (**B**,**C**,**E**–**H**) Graphs present mean ± SD (*n* = 1–4); * *p* < 0.05 assessed using one-way ANOVA.

To investigate the effects of PC on SKPs, we isolated SKPs from young (SNS < 5%) and old (SNS > 15%) primary fibroblast cultures using a standardized low-pH SKP isolation protocol [35]. SKPs were cultured and treated with 0.1% or 0.5% PC for 5 days in SKP-specific growth medium. After treatment, we assessed SKP morphology, growth capacity, and the expression of stem cell markers. Untreated SKPs, isolated from early passage (young) fibroblast cultures, formed small, uniform spheroid and typically maintaining a healthy phenotype (Figure 4A–C) [35,53]. In contrast, untreated SKPs from aged (old passages) fibroblast cultures, showed reduced spheroid numbers and exhibited significantly larger diameters due to increased fusion events, characteristic of aged SKPs with compromised cellular health (Figure 4A–C) [53]. Notably, PC treatment at both 0.1% and 0.5% restored the aged SKP spheroids’ morphology, reducing the spheroid size to approximately 100–120 µm, comparable to the untreated young SKP spheroids (Figure 4A,B), indicating improved cellular integrity. Hence, the PC treatment enhanced the expansion and growth of old SKPs (Figure 4A–C).

In addition to SKPs, we investigated the effects of PC treatment on hMSCs (umbilical) and BMSCs (bone) (Figure 4D–H). Mesenchymal stem cells (MSCs) are multipotent stem cells capable of differentiating into mesenchymal lineages, highly self-renewing and widely distributed throughout the body, playing a crucial role in tissue regeneration [54]. BMSCs represent a bone marrow-resident stem cell population with similar regenerative potential and expression of specific stemness markers [55]. Both hMSCs and BMSCs were cultured as spheroids in SKP growth medium and treated for 5 days with 0.1% and 0.5% PC, alongside untreated (mock) controls. Mock-treated hMSC spheroids were smaller than SKPs, with an average diameter of 54 µm, at day 5 (Figure 4D,E). PC treatment enhanced both the size and number of hMSC spheroids, with PC 0.5% increasing hMSC spheroid size by 9.9%, suggesting improved proliferation (Figure 4D–F). Untreated BMSC spheroids displayed an average diameter of approximately 206 µm (Figure 4G). Treatment with 0.1% PC resulted in a 15.7% increase in spheroid diameter accompanied by an increase in spheroid number (Figure 4G,H), indicating enhanced proliferative capacity and growth. Collectively, these results demonstrate that PC treatment supports the expansion, morphology, and maintenance of multiple adult stem cell types.

To elucidate whether the observed enhancements in stem cell growth and morphology were accompanied by changes in stem cell identify, we examined the expression of key genes associated with stemness. The expression levels of stemness markers including Sox2, Oct4, Nanog, TG30, Nestin, and Vimentin were quantified in SKPs, hMSCs, and BMSCs using qPCR and immunocytochemistry. This approach facilitated a comprehensive evaluation of whether PC treatment influences stem cell properties.

**Figure 5:**
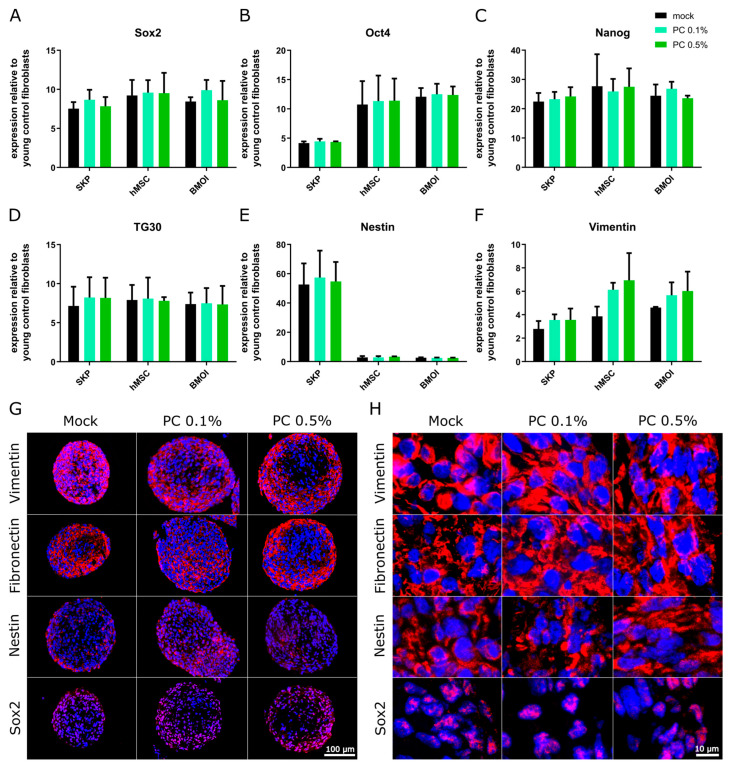
Stem cell marker profile in SKPs, hMSCs, and BMSCs at day 5 of treatment with and without PC treatment relative to young control fibroblasts. Panel (**A**–**F**) despite the quantitative real-time PCR analysis of Sox2 (**A**), Oct4 (**B**), Nanog (**C**), TG30 (**D**), Nestin (**E**), and Vimentin (**F**) in SKPs, hMSCs, and BMSCs. The relative expression ratio was normalized to expression of GAPDH. Graphs show mean ± SD (*n* = 3), using two-way ANOVA. (**G**,**H**) Representative merged images of immunocytochemistry for Vimentin, fibronectin, nestin, and SOX2 in BMSCs (red). Cell nuclei are counterstained with DAPI (blue). Scale bar 100 µm (**G**) and 10 µm (**H**). Cells were counterstained with DAPI.

Mock-treated SKPs, hMSCs, and BMSCs exhibited an elevated expression of all analyzed stem cell markers (Sox2, Oct4, Nanog, TG30, Nestin, and Vimentin) compared to fibroblast as controls (Figure 5A–F), confirming the maintenance of stem cell properties in these cultures. The treatment with PC at both 0.1% and PC 0.5% concentrations did not significantly alter the expression of Sox2, Oct4, Nanog, TG30, Nestin, or Vimentin across all three stem cell types, with expression maintained comparable to that of the mock-treated groups (Figure 5A–F). These findings suggest that PC treatment does not disrupt the transcriptional profile associated with the stemness of dermal SKPs, hMSCs, and BMSCs.

To further validate these results, immunofluorescence staining was performed on SKP, hMSC, and BMSC spheroid sections using specific antibodies against vimentin, fibronectin, nestin, and SOX2 (Figure 5G,H, Supplementary Figure S2). Figure 5H illustrates the characteristic localization and signals of these markers, consistent with the gene expression data obtained from qPCR results. In all stem cell models, PC treatment at both 0.1% and 0.5% concentrations did not lead to any significant changes in protein expression levels of these stem cell markers (Figure 5G,H, Supplementary Figure S2).

Overall, these findings indicate that PC treatment supports the growth, maintenance, and proliferation of adult stem cells without compromising their stemness or altering the expression of key stem cell markers, indicating its potential positive effect on preserving stem cell identity.

### 3.4. Anti-Aging Effect of PC

**Figure 6:**
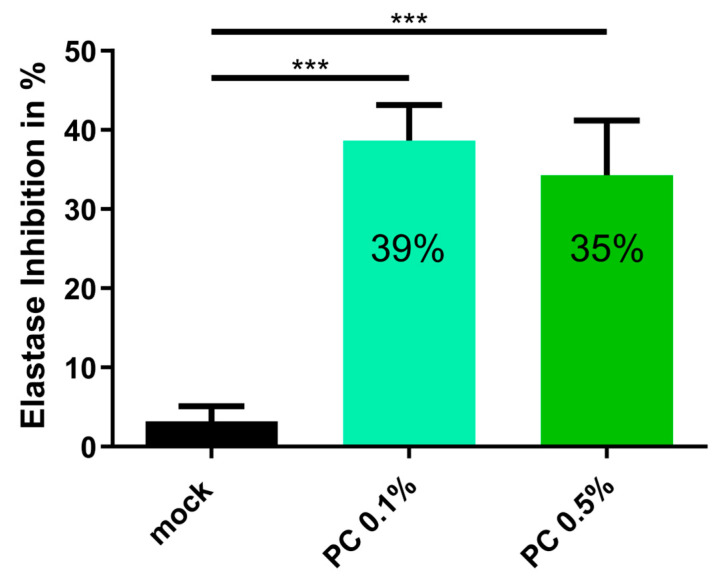
Elastase inhibitory effect of the PC extract. The PC extract was prepared at concentrations of 0.1% and 0.5% in Tris-HCl buffer, whereas the control blank consisted solely of Tris-HCl buffer. For statistical analysis, both PC treatments were compared to the mock control group, which consisted of Tris-HCl buffer without extract. The assay was performed in three technical replicates. Graphs present mean ± SD (*n* = 3); *** *p* < 0.001; assessed using one-way ANOVA.

To complement the cellular and molecular analyses, we next evaluated the functional anti-aging potential of PC through a skin relevant enzymatic assay. During aging, elastase activity increases, leading to the enhanced degradation of dermal elastin, which contribute to reduced skin elasticity and visible signs of skin aging [56]. To assess whether PC exhibits anti-aging properties, an elastase inhibition assay was performed using both 0.1% and 0.5% PC concentrations. The PC extract was dissolved in Tris-HCl buffer, while the control blank consisted of Tris-HCl buffer without the extract. Tris-HCl buffer was used as a negative control and showed no elastase inhibition, confirming the specificity of the inhibitory effect observed with PC. For statistical analysis, Tris-HCl buffer without extracts was used as a mock control group. The results demonstrated that PC effectively inhibited elastase activity compared to the mock treatment, with 0.1% PC reducing enzymatic activity by 39% and 0.5% PC by 35% (Figure 6). These results indicate that PC possesses elastase-inhibitory properties, suggesting its potential to preserve extracellular matrix integrity and mitigate skin aging.

## 4. Discussion

Aging is a complex biological process that affects cellular homeostasis and tissue function, with skin aging being a visible manifestation. This study investigated the effects of the argan extract PhytoCellTec™ Argan (PC) on human skin fibroblasts and adult stem cells. The PC treatment significantly enhanced fibroblast proliferation, as demonstrated by an increased proportion of BrdU-positive cells and cumulative population doublings while reducing senescence levels. The most pronounced effects were observed at concentrations of 0.1% and 0.5%, indicating an optimal range for promoting cellular proliferation and reducing cellular senescence. These findings align with previous studies showing that plant-derived bioactive compounds can stimulate fibroblast activity and reduce senescence, thereby supporting skin regeneration and wound healing [57,58,59]. Bejaoui et al. similarly reported that argan press cake extract promoted proliferation and reduced senescence in dermal papilla cells, a specialized fibroblast population involved in hair follicle health [60].

Interestingly, higher concentrations of the PC extract (1% and 5%) induced adverse effects. At 1%, PC increased senescence by 6%, while 5% resulted in complete cell death within 24 h. This underscores a dose-dependent response, where low to moderate concentrations exert beneficial effects, but higher doses may provoke cellular stress, apoptosis, or metabolic imbalance. These effects are consistent with previous observations that phytochemicals such as phytosterols, tocopherols, and saponins, components of argan extracts, exhibit anti-proliferative effects on cancer cell lines at high doses [61,62].

The aging process is closely associated with oxidative stress, characterized by an imbalance between ROS production and the antioxidant defense system [63,64]. Excess ROS damages cellular components such as lipids, proteins, and DNA, contributing to cellular dysfunctions and the breakdown of collagen and elastin, leading to reduced elasticity and aging of the skin [65,66]. Antioxidants are therefore widely studied for their ability to counteract oxidative stress and delay the aging process [67]. In this study, aged fibroblasts characterized by elevated basal ROS levels were used to evaluate the antioxidant effects of PC. The aim was to determine whether PC could help restore redox homeostasis toward physiological levels. The observed reductions in ROS levels by 7.1% and 16.6% following treatment with PC 0.1% and 0.5%, respectively, indicate a meaningful modulation of oxidative stress in this cellular context. These results suggest that PC contributes to the normalization of intracellular ROS levels, supporting its role in mitigating oxidative damage during aging. Although a classical antioxidant control was not included in this study, its incorporation in future experiments will be important for benchmarking the antioxidant efficacy of PC against established reference compounds. PC exhibited strong antioxidant activity, demonstrated by ABTS/TEAC scavenging and reduced intracellular ROS levels at 0.1% and 0.5%. These effects are likely due to bioactive compounds in the argan callus extract, such as linoleic and oleic acids, polyphenols, sterols, and tocopherols, which have documented antioxidant activity [23,68,69]. PC also inhibited elastase activity, which increases during aging and contributes to elastin degradation and the loss of skin elasticity [70,71,72]. Inhibiting elastase helps preserve the dermal extracellular matrix, suggesting a functional anti-aging benefit that complements PC’s antioxidant action.

Autophagy is a fundamental process for maintaining cellular integrity, particularly under conditions of oxidative stress. It enables the degradation and recycling of damaged organelles and macromolecules, thus preserving cell function and survival [73,74]. However, autophagic efficiency declines with age. PC treatment enhanced autophagy, as indicated by increased autophagosome formation (LC3B II/I ratio) and decreased p62 levels. These results suggest improved autophagy flux and the clearance of damaged cellular components. Similar autophagy-promoting effects have been observed with argan oil in 7-ketocholesterol–stressed oligodendrocytes, and for other plant extracts such as *Crepidiastrum denticulatum*, lotus, and rosemary in fibroblasts [75,76,77].

Autophagy is also tightly linked to cellular energy status. PC treatment increased ATP production, suggesting improved mitochondrial function and energy metabolism. Since ATP is essential for cellular repair and regeneration, this increase may support skin renewal and tissue integrity. Moreover, PC activated AMPK, a key energy sensor that promotes ATP production and regulates metabolism under stress conditions [78,79]. By modulating ROS, autophagy, and energy metabolism, PC appears to promote cellular homeostasis, especially under aging-associated stress.

In addition to its antioxidant and metabolic effects, PC modulated inflammatory signaling. Chronic low-grade inflammation, often driven by NFκB activation, is a hallmark of skin aging and a major contributor to the senescence-associated secretory phenotype (SASP) [4,52,80]. PC treatment significantly reduced NFκB activation and the expression of senescence markers p16 and p21, suggesting a dual anti-inflammatory and anti-senescence effect. These results align with findings from other botanical compounds such as grape polyphenols, *Astragalus* extracts, and *Salvia haenkei*, all of which exhibit anti-inflammatory and anti-senescent properties [81,82,83]. Argan oil’s anti-inflammatory effects have also been shown in vivo, demonstrating comparable efficacy to diclofenac in a rat inflammation model [84].

Beyond its effects on fibroblasts, PC exerted significant benefits on adult stem cells, which are essential for maintaining skin regeneration throughout life. The integrity of a stem cell niche is critical for preserving stem cell function during aging [85,86,87]. PC treatment enhanced the growth and morphology of skin-derived precursor cells (SKPs) and mesenchymal stem cells (MSCs), while preserving the expression of key stemness markers (Sox2, Oct4, nanog, TG30, nestin, and vimentin). This suggests that PC supports proliferation and regenerative function without compromising stem cell identity. Similar results have been observed with other botanical extracts, such as *Rosa roxburghii* and *Withania somnifera*, which promote telomerase activity, stem cell growth, and reduced senescence [88,89,90]. Maintaining healthy and functional stem cells is crucial for preventing age-related decline in skin elasticity and repair capacity [91,92,93].

PCs also demonstrated functional anti-aging activity through elastase inhibition. Since elastase degrades elastin fibers, contributing to the loss of skin elasticity, its inhibition by PC supports extracellular matrix preservation. These effects, in combination with PC’s antioxidative and anti-inflammatory properties, suggest a multifaceted mechanism for improving skin resilience and structure.

The observed effects of PC on oxidative stress, autophagy, inflammation, and cellular senescence suggest a multifaceted mechanism of action involving key molecular signaling pathways. One potential mechanism involves the activation of AMP-activated protein kinase (AMPK), a master regulator of cellular energy status, which promotes mitochondrial biogenesis, enhances autophagy, and suppresses inflammation [94]. PC-induced AMPK activation may contribute to increased ATP production and autophagy flux, as reflected by elevated LC3B-II/I ratios and decreased p62 levels. Concurrently, the reduction in ROS levels implies that PC may contain bioactive antioxidant compounds, such as polyphenols and tocopherols, that directly scavenge free radicals and indirectly activate cytoprotective transcription factors like Nrf2 [95,96,97]. Additionally, the suppression of NFκB phosphorylation suggests that PC interferes with inflammatory signaling, through AMPK-dependent and redox-sensitive mechanisms. These pathways may converge to delay cellular senescence and maintain stem cell and fibroblast function. Further studies are needed to identify the specific active compounds within PC responsible for these effects and to validate their roles in modulating these interconnected pathways.

Although our study provides strong in vitro evidence for the anti-aging potential of PC, further in vivo studies are required to confirm its effects under physiological conditions and to evaluate long-term outcomes. In addition, the specific bioactive components within the PC extract remain to be identified, and future research should include a detailed phytochemical analysis to link composition with biological activity. While our assays demonstrated consistent improvements in oxidative stress, proliferation, senescence, and stem cell function, some were performed without classical positive controls, such as established antioxidants or reference anti-aging compounds. This may limit the direct comparison of PC’s efficacy to standard benchmarks. However, given the focus of this study on characterizing PC’s effects specifically in aged fibroblasts and stem cells, the absence of such controls does not diminish the internal consistency of our findings. Future studies incorporating well-defined positive controls, alongside in vivo validation and phytochemical profiling, will be important to further substantiate the therapeutic potential of PC.

Collectively, our findings indicate that PC modulates multiple hallmarks of aging—oxidative stress, energy metabolism, autophagy, inflammation, and stem cell function—through coordinated molecular and cellular mechanisms, supporting its potential as a plant-derived strategy to counteract age-related cellular decline.

## 5. Conclusions

This study demonstrates that PhytoCellTec™ Argan (PC) exerts anti-aging effects in vitro by targeting multiple cellular processes relevant to skin aging. PC reduced oxidative stress, enhanced mitochondrial ATP production, improved autophagic efficiency, and attenuated inflammatory and senescence-associated signaling in human dermal fibroblasts. Additionally, PC supported the growth and maintenance of adult stem cells without altering their stemness profile. These findings suggest that PC contributes to the preservation of cellular homeostasis and regenerative capacity in human skin cells, providing a foundation for further investigation in physiological and clinical settings.

## Figures and Tables

**Table 1:** Used cell strains with respective passage numbers and senescence levels.

Cell Strain	Passage Number	Senescence
GM05757C	Passage 9–24	4.1–25.0%
GM05565A	Passage 10–23	4.4–24.6%
HGFDFN369	Passage 7–21	3.1–23.7%
GM01651C	Passage 11–21	5.0–19.5%

## Data Availability

Data are contained within the article and Supplementary Materials.

## Supplementary Materials

The following supporting information can be downloaded at: https://www.mdpi.com/article/10.3390/antiox14070804/s1, Figure S1: Determination of working concentration of PhytoCell^TM^; Figure S2: Stem cell marker profile in SKPs, hMSCs, and BMSCs at day 5 of treatment with and without PC treatment relative to young control fibroblasts; Figure S3: Full-length scan of Western blots from Figure 2; Figure S4: Full-length scan of Western blots from Figure 3; Table S1: Primer sequences used for qPCR.

## Abbreviations

The following abbreviations are used in this manuscript:

AMPK	AMP-activated protein kinase
ATP	Adenosine triphosphate
BrdU	5-Bromo-2′-deoxyuridine
BMSC	Bone marrow stem cell
CPD	Cumulative population doubling
DCF	Dichlorofluorescein
DCFDA	2′,7′-dichlorofluorescin diacetate
hMSC	Human mesenchymal stem cell
MDC	Monodansylcadaverine
OCT	Optimal cutting medium
PC	PhytoCellTec™ Argan
ROS	Reactive oxygen species
SASP	Senescence-associated secretory phenotype
SNS	Senescence
SKP	Skin-derived precursor cell
TEAC	TROLOX Equivalent Antioxidant Capacity
TLA	Three-letter acronym
LD	Linear dichroism
