# Supplementary material for: Argan Callus Extract Restores Skin Cells via AMPK-Dependent Regulation of Energy Metabolism, Autophagy, and Inflammatory Pathways
Source: Antioxidants (Basel). 2025 Jun 28;14(7):804. doi: 10.3390/antiox14070804 (PMC12291937; doi:10.3390/antiox14070804)
Supplement: Supplementary file 1 [file antioxidants-14-00804-s001.zip › antioxidants-3662840-supplementary.pdf]

Supplementary Figures

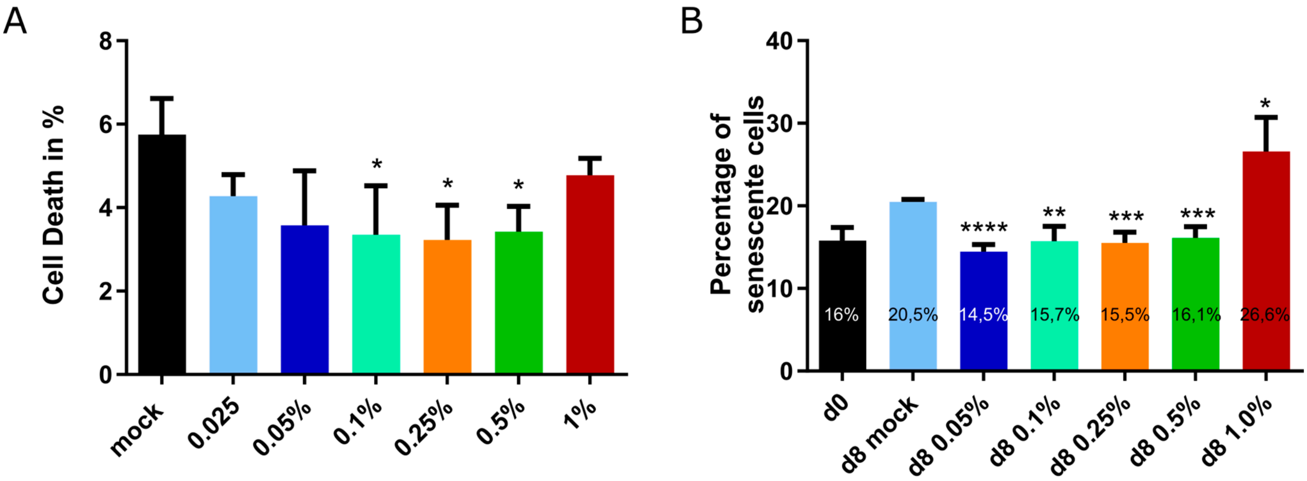

Figure S1: Determination of working concentration of PhytoCell. Fibroblasts were treated for 8 days with and without PhytoCell extract. (A) Percentage of dead cells. (B) Percentage of senescent cells at day 8 of treatment. (A,B) Values are presented as mean ± SD ($n = 3$); * $p < 0.05$; ** $p < 0.01$; *** $p < 0.001$; **** $p < 0.0001$; assessed using one-way ANOVA.

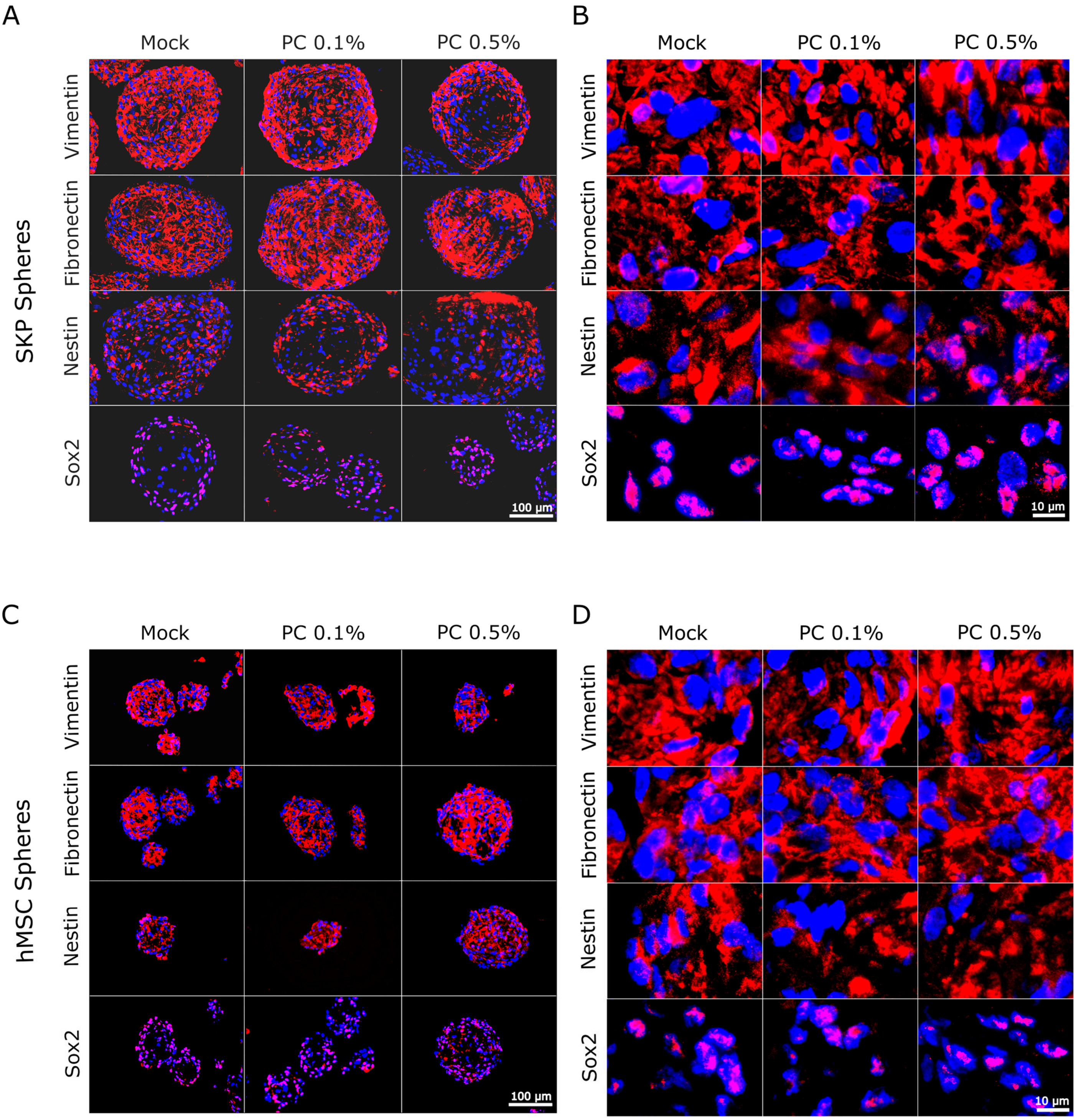

Figure S2: Stem cell marker profile in SKPs and hMSCs, and BMSCs at day 5 of treatment with and without PC treatment relative to young control fibroblasts. (A)-(D) Representative merged images of immunocytochemistry for Vimentin, fibronectin, nestin and SOX2 in SKPs and hMSCs (red). Scale bar 100 µm (A), (C) and 10 µm (B), (D). Cells were counterstained with DAPI (blue).

Full-lenght scans of western blots in Figure 2

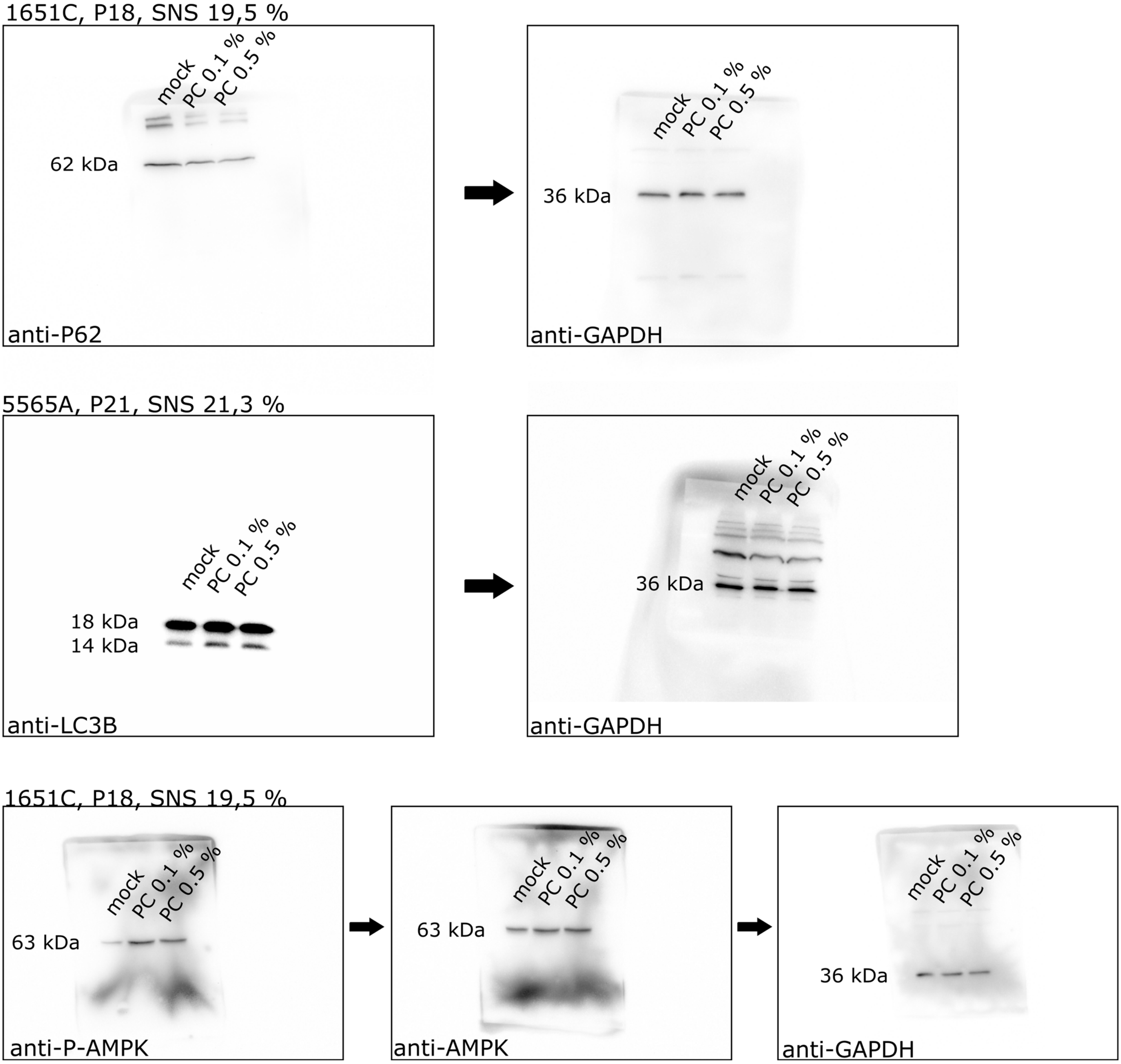

Figure S3: Full-length scan of western blots from Figure 2. Western blots of control cells (GM05565A, GM01651C) probed with anti-P-P62 (left scan), anti-LC3B (left scan), anti P-AMPK (left scan), anti-AMPK (middle scan), and anti-GAPDH (right scan)

Full-lenght scans of western blots in Figure 2

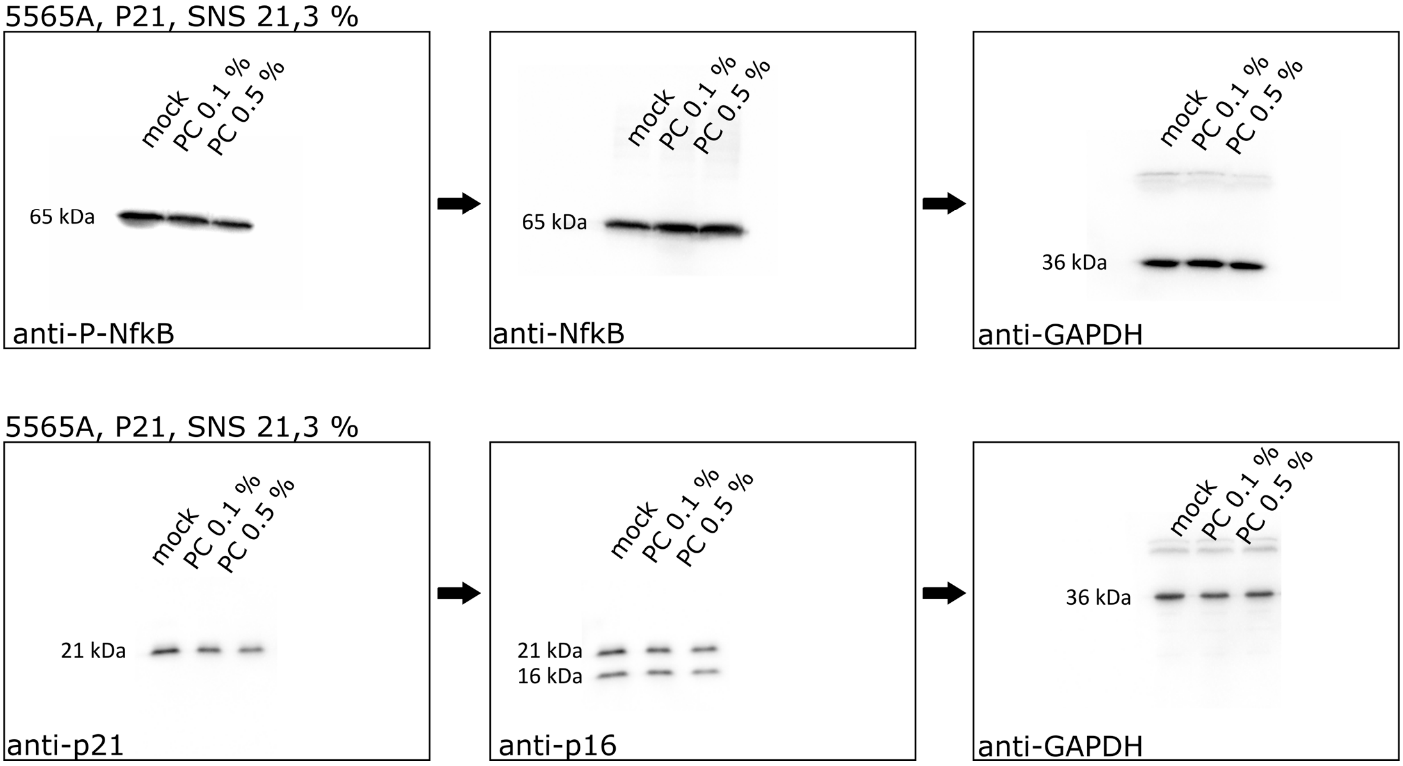

Figure S4: Full-length scan of western blots from Figure 2. Western blots of control cells (GM05565A) probed with anti-P-NfκB (left scan), anti-NfκB (middle scan), anti p-16 (left scan), anti-p21 (middle scan), and anti-GAPDH (right scan)

Table S1: Primer sequences used for qPCR

Primer sequence Target	Gene
FW: 5′- GCTACAGCATGATGCAGGACCA -3′<br>REV: 5′- TCTGCGAGCTGGTCATGGAGTT -3′	Sox2
FW: 5′- CCTGAAGCAGAAGAGGATCACC -3′<br>REV: 5′- AAAGCGGCAGATGGTCGTTTGG -3′	Oct4
FW: 5′- CAGAAGGCCTCAGCACCTAC -3′<br>REV: 5′- GCCTCCAAGTCACTGGCAG -3′	Nanog
FW: 5′- TCGCCATTGAAATAGCTGCGGC -3′<br>REV: 5′- CGCATAGTGGATGGCTTTCAGC -3′	TG30
FW: 5′- TCAAGATGTCCCTCAGCCTGGA -3′<br>REV: 5′- AAGCTGAGGGAAGTCTTGGAGC -3′	Nestin
FW: 5′- AGGCAAAGCAGGAGTCCACTGA -3′<br>REV: 5′- ATCTGGCGTTCCAGGGACTCAT -3′	Vimentin
FW: 5′- GTCTCCTCTGACTTCAACAGCG -3′<br>REV: 5′- ACCACCCTGTTGCTGTAGCCAA -3′	GAPDH
